# Gut Microbiota and Its Metabolites Modulate Pregnancy Outcomes by Regulating Placental Autophagy and Ferroptosis

**DOI:** 10.3390/antiox14080970

**Published:** 2025-08-07

**Authors:** Xingyu Du, Mabrouk Elsabagh, Feiyang He, Huisi Wu, Bei Zhang, Kewei Fan, Mengzhi Wang, Hao Zhang

**Affiliations:** 1 Laboratory of Metabolic Manipulation of Herbivorous Animal Nutrition, College of Animal Science and Technology, Yangzhou University, Yangzhou 225009, China; mx120240920@stu.yzu.edu.cn (X.D.); 221902307@stu.yzu.edu.cn (F.H.); 231902320@stu.yzu.edu.cn (H.W.); mz120231562@stu.yzu.edu.cn (B.Z.); 2 Key Laboratory of Fujian Universities Preventive Veterinary Medicine and Biotechnology, Longyan University, Longyan 364012, China; 3 Department of Animal Production and Technology, Faculty of Agricultural Sciences and Technologies, Niğde Ömer Halisdemir University, Nigde 51240, Turkey; 4 College of Agriculture, University of Al Dhaid, Al Sidra, Al Dhaid, Sharjah P.O. Box 27272, United Arab Emirates; 5 State Key Laboratory of Sheep Genetic Improvement and Healthy Production, Xinjiang Academy of Agricultural Reclamation Science, Shihezi 832000, China

**Keywords:** autophagy, ferroptosis, gut microbiota, placental development, pregnancy outcomes

## Abstract

During pregnancy, the regulation of autophagy and ferroptosis dynamically supports placental development and fetal health. Both processes—autophagy, clearing damaged organelles to maintain placental function, and ferroptosis, driven by iron-dependent lipid peroxidation—are involved in pathological conditions such as preeclampsia. Emerging evidence suggests that gut microbiota-derived metabolites act as key regulators of this balance, yet their specific roles across different trimesters remain unclear. This review compiles evidence on how gut microbiota metabolites, like short-chain fatty acids and trimethylamine N-oxide, serve as trimester-specific modulators of the autophagy–ferroptosis balance during pregnancy. We explain how these metabolites influence pregnancy outcomes by regulating placental autophagy and ferroptosis. Furthermore, we explore potential diagnostic and therapeutic approaches for pregnancy complications, focusing on metabolite-based biomarkers and interventions that target microbial–metabolic interactions.

## 1. Introduction

Ferroptosis, an iron-dependent cell death process, is characterized by intracellular iron overload, excessive production of reactive oxygen species (ROS), and lipid peroxidation [1]. Emerging evidence highlights its physiological regulating roles such as suppressing tumor progression and mitigating neurodegenerative pathologies. However, its regulatory mechanisms in pregnancy remain poorly understood, particularly in placental development and gestational complications such as preeclampsia (PE) and fetal growth restriction (FGR) [2,3].

Autophagy, a conserved lysosome-dependent degradation process, primarily sustains cellular homeostasis by recycling damaged organelles and misfolded proteins into reusable metabolites such as amino acids [4,5,6]. While basal autophagy mitigates oxidative stress by scavenging ROS-generating components, excessive activation of lipophagy and ferritinophagy promotes ferroptosis via enhancing iron release and lipid peroxidation. This intricate crosstalk underscores the context-dependent interplay between autophagy and ferroptosis, which may dictate pregnancy outcomes [7]. Despite a maternal mortality rate of 5–20% linked to gestational disorders, therapeutic strategies targeting these pathways remain underdeveloped [8]. Notably, various studies have demonstrated the effect of placental autophagy and ferroptosis during pregnancy, which gives new potential treatment and drug targets for pregnancy complications.

The gut microbiota often serves as the “second genome”, which profoundly influences host physiology through metabolite production. For instance, microbiota-derived trimethylamine N-oxide (TMAO) may exacerbate placental ferroptosis by amplifying lipid peroxidation, whereas short-chain fatty acids (SCFAs) could enhance autophagy-mediated cytoprotection [9]. Intriguingly, microbial dysbiosis is implicated in adverse pregnancies; its regulatory effects on placental autophagy and ferroptosis are largely unexplored. Thus, exploring how gut microbiota regulates placental autophagy and ferroptosis during pregnancy may yield novel biomarkers and microbiota-targeted interventions to improve gestational health.

## 2. Gut Microbiota–Pregnancy Axis: A Central Regulator of Pregnancy Homeostasis

The gut microbiota–pregnancy axis has emerged as a prominent focus in current biomedical research. Accumulating evidence demonstrates that gut microbiota and its metabolites exert significant regulatory effects on maternal physiology and fetal development during pregnancy. These microbial communities influence immune modulation, metabolic adaptations, and inflammatory responses essential for a healthy gestational process. Concurrently, advancements in diagnostic and therapeutic approaches targeting the gut microbiota during pregnancy are progressively being explored, aiming to improve maternal and neonatal outcomes. Moreover, autophagy and ferroptosis, as two distinct, regulated forms of cell death, have been identified as critical modulators of pregnancy outcomes. Autophagy contributes to trophoblast differentiation and placental homeostasis, while dysregulation of ferroptosis has been implicated in pregnancy complications, such as preeclampsia and recurrent pregnancy loss. Understanding the interplay between these cell death pathways and the gut microbiota may offer novel insights into the mechanisms underlying pregnancy maintenance and disorders. The gut microbiota harbors a collective genome, referred to as the microbiome, which is estimated to be approximately 150 times larger than the host genome. The gut microbiome encodes a vast array of enzymatic proteins and metabolic functions that are absent in the host’s genome, significantly expanding the host’s metabolic capabilities. These microbial genes critically regulate metabolic processing, hormone regulation, and immune modulation during pregnancy [9,10]. As a dynamic interface between the host and external environment, the gut microbiota colonizing the intestinal lumen plays a pivotal role in translating environmental signals into host physiological functions. Environmental perturbations, such as dietary changes, antibiotic exposure, or stress, frequently induce microbial dysbiosis, disrupting homeostatic equilibrium of the gut ecosystem and predisposing the host to systemic pathophysiology conditions [11]. Through fermentation of residual dietary substrates, gut microbiota synthesizes essential micronutrients, including B vitamins and produces bioactive compounds critical for maintaining gestational physiology [12]. Notably, SCFAs exert functions beyond energy provision: they enhance gastrointestinal barrier integrity via upregulating tight junction proteins such as occludin and attenuate proinflammatory cytokine cascades induced by immune axis imbalance [13]. Dysbiosis triggered by external factors such as dietary shifts and antibiotic exposure, as well as endogenous stressors including psychological strain and oxidative damage, manifests as alterations in microbial community profile and metabolic pathways. These disturbances increase intestinal permeability, facilitating microbial translocation and leakage of metabolites into systemic circulation [14]. Recent debates have centered on the existence of placental microbial colonization, with two contrasting perspectives emerging. One hypothesis suggests that compromised intestinal barrier integrity facilitates ectopic migration of microbes to placental niches. In contrast, opposing views emphasize that the placenta remains sterile under physiological conditions. The gut microbiota–metabolites axis exerts dual regulatory effects on pregnancy progression. Beneficial metabolites, such as SCFAs and indole derivatives, attenuate placental inflammatory responses primarily by inhibiting the NF-κB pathway. Conversely, elevated levels of certain bile acid derivatives—specifically deoxycholic acid (DCA) and chenodeoxycholic acid (CDCA)—have been shown to induce trophoblast apoptosis through mechanisms involving mitochondrial dysfunction and endoplasmic reticulum stress pathways [15]. Clinical studies consistently report associations between microbial dysbiosis and pregnancy complications including PE and gestational diabetes mellitus (GDM), highlighting the diagnostic and therapeutic potential of targeting the gut microbiota in these conditions [9,16,17].

In conclusion, the gut microbiota–pregnancy axis stands at the forefront of current biomedical research, reflecting its pivotal role in maternal and fetal health. Accumulating evidence demonstrates that microbial metabolites such as SCFAs, indole derivatives, and bile acid metabolites act as master regulators of gestational processes. These metabolites influence immune modulation, metabolic adaptation, and the maintenance of placental and fetal homeostasis, making them promising candidates for diagnostic biomarkers and targets for microbiota-based therapies. Recent studies suggest that the interplay between autophagy and ferroptosis, two regulated cell death pathways governed by microbial metabolites, is intricately modulated by microbial metabolites. This autophagy–ferroptosis crosstalk serves as a critical checkpoint for placental development and fetal programming. Microbial metabolites can modulate these pathways by upregulating protective autophagic responses as well as regulating iron metabolism and lipid peroxidation, thereby influencing ferroptosis susceptibility. However, the interconnected network involving autophagy, ferroptosis, and microbial signaling remains underexplored. Unraveling these complex interactions offers new insights into the pathogenesis of pregnancy disorders such as preeclampsia and gestational diabetes and yields novel treatments to optimize pregnancy outcomes and long-term offspring health.

## 3. Autophagy and Ferroptosis: The Basic Molecular Mechanisms

### 3.1. Core Mechanisms of Autophagy

**Figure 1:**
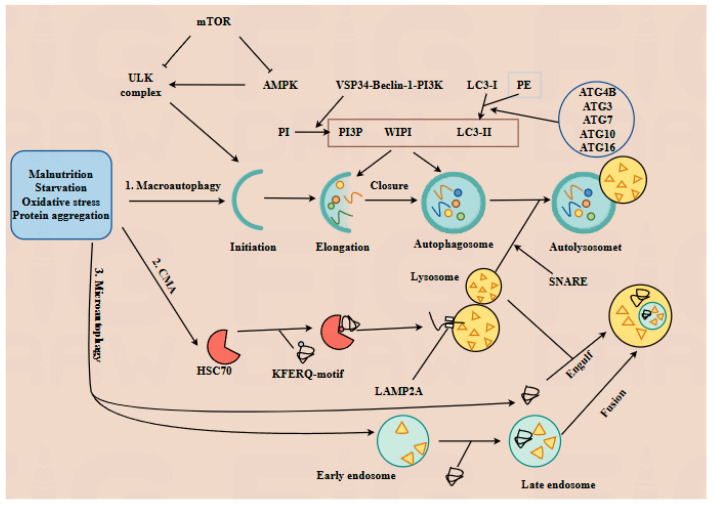
The mechanisms of three types of autophagy. Autophagy, a conserved lysosomal degradation pathway, is classified into three types—macroautophagy, chaperone-mediated autophagy (CMA), and microautophagy—each activated by distinct stressors, including nutrient deprivation, oxidative stress, and protein aggregation. 1. Macroautophagy proceeds through five sequential stages. 1.1. Initiation: Nutrient deprivation triggers AMP-activated protein kinase (AMPK)-mediated mTOR inactivation, promoting the assembly of ULK complexes. 1.2. Elongation and closure: The Vps34-Beclin-1-PI3K complex promotes phosphorylation of phosphatidylinositol (PI) into PI3P (phosphatidylinositol 3-triphosphate). Microtubule-associated protein 1 light chain 3 (LC3-I) binds to phosphatidylethanolamine, and its conversion to LC3-II is facilitated by ATG4B, ATG3, ATG7, ATG10, and ATG16. Recruitment of WIPI proteins promotes autophagosome formation. 1.3. Fusion and degradation: SNARE proteins mediate lysosome–autophagosome fusion to form autolysosomes, where targeted substrates are degraded into amino acids by lysosomal acid proteases. 2. CMA relies on chaperone proteins such as heat-shock cognate protein 70 (HSC70), which deliver target proteins containing KFERQ-like motifs to lysosome-associated membrane protein type 2A (LAMP2A). Following HSC70-LAMP2A binding, substrates are translocated into the lysosome and degraded into amino acids. 3. Microautophagy can be divided into two subtypes: 3.1. Lysosome-dependent microautophagy: Lysosomes directly engulf cytoplasmic components. 3.2. Endosome-mediated microautophagy: Substrates are invaginated into early endosomes. After maturation into late endosomes, they entirely fuse with lysosomes, inducing the degradation of the late endosomes.

Autophagy is an evolutionarily conserved process that degrades the long-lived proteins and damaged organelles into amino acids to maintain cellular homeostasis, serving as a key regulatory mechanism in cell physiology [18]. Autophagy is classified into three types based on how substrates are delivered to lysosomes: macroautophagy, microautophagy, and chaperone-mediated autophagy (CMA); all three are activated by stress conditions including malnutrition, starvation, oxidative stress, and protein aggregation [19]. The distinct molecular mechanisms of these three autophagy types are illustrated in Figure 1. Overall, autophagy regulation is complex and multifaceted, but its specific mechanisms remain incompletely understood.

This article primarily reviews the role of autophagy in regulating physiological and pathological processes during embryonic and fetal development. It further explores how gut microbiota and its metabolites influence embryonic and fetal development by modulating autophagy (Figure 1).

### 3.2. Core Mechanisms of Ferroptosis

Ferroptosis is an iron-dependent cell death pathway characterized by distinct morphological and biochemical features, involving the alteration or disappearance of mitochondrial cristae, reduction in mitochondrial volume, increased membrane density, along with elevated levels of ROS lipid peroxidation, and divalent iron content [20,21]. A multitude of substrates play a crucial role in positively regulating ferroptosis. For instance, phospholipid hydroperoxides (PLOOH) are essential substrates for initiating ferroptosis. PLOOH can react with Fe(II) or Fe(III) generating lipid alkyl radicals and lipid peroxyl radicals, which can further react with polyunsaturated fatty acids (PUFA), promoting the accumulation of additional PLOOH. Once the accumulation of PLOOH exceeds a critical threshold, ferroptosis is triggered in the cell. Subsequently, the damage to the cell’s plasma membrane rapidly becomes irreversible, leading to cell death [22]. ROS can facilitate lipid oxidation, which in turn accelerates the degradation of ferroportin (FPN) and ultimately leads to cell death [23]. Long-chain acyl-coenzyme A synthase 4 (ACSL4) catalyzes the esterification of specific PUFA, such as arachidonic acid (20:4) and adrenic acid (22:4), to coenzyme A, generating the corresponding acyl-CoA. These acyl-CoAs are subsequently incorporated into phospholipids, promoting their peroxidation. This phospholipid peroxidation is a key event that triggers ferroptosis, a form of iron-dependent cell death [24]. Iron serves as a critical component at the active site of lipoxygenase enzymes (LOXs), facilitating PUFA into PLOOH within the cell membrane, which can further induce ferroptosis. In addition to being transported into the cell via the transferrin pathway, extracellular iron can also be endocytosed into the cell and subsequently enter early endosomes. Subsequently, DMT1 on the endosomal membrane further transports FE (II) into the cytoplasm, where it can be stored as ferritin or delivered to organelles such as mitochondria for protein synthesis [25,26]. Additionally, the accumulation of free iron within cells can lead to increased production of ROS via the Fenton and Haber–Weiss reactions. Iron serves as a lipid peroxidation catalyst, facilitating the oxidation of lipids [27]. A multitude of substrates play a crucial role in negatively regulating ferroptosis. A variety of antioxidant substances, including glutathione peroxidase 4 (GPX4), vitamin E, CoQ10H2, NADPH, and ferroptosis suppression protein 1 (FSP1), play crucial roles in mitigating lipid peroxidation and scavenging ROS, thereby reducing the cell’s sensitivity to ferroptosis [28].

**Figure 2:**
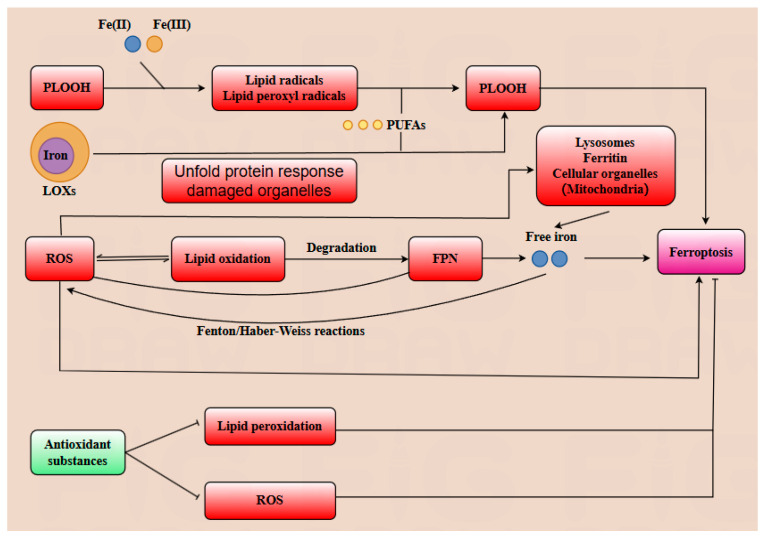
Ferroptosis is hallmarked by lipid peroxidation, excessive reactive oxygen species (ROS) generation, and iron overload. Phospholipid hydroperoxides (PLOOH) react with free iron to generate lipid alkyl radicals and lipid peroxyl radicals, which further promote the oxidation of polyunsaturated fatty acids (PUFAs) to PLOOH. Iron serves as a critical cofactor for lipoxygenases (LOXs), which catalyze PUFAs oxidation to PLOOH, accelerating ferroptosis progression. Additionally, the unfolded protein response and damaged organelles (e.g., mitochondria) augment ROS production, which reinforces lipid oxidation. This cascade induces the degradation of ferroportin (FPN), a key iron exporter; consequently, elevated free Fe^2+^ levels further fuel ferroptosis via Fenton and Haber–Weiss reactions, generating additional ROS. Notably, free iron is released from iron stores in organelles, including ferritin, mitochondria, and lysosomes, exacerbating iron overload. In contrast, cellular antioxidants suppress lipid peroxidation and ROS generation, thereby inhibiting ferroptosis.

In conclusion, the regulatory mechanisms of ferroptosis are multifaceted and intricate. Emerging evidence highlights the pivotal role of ferroptosis in modulating diverse cellular biological processes, particularly in suppressing tumor cell proliferation and expansion. This review aims to comprehensively elucidate the role of ferroptosis in pregnancy and delineate the regulatory mechanisms of gut microbiota and its metabolites on ferroptosis during gestation (Figure 2).

### 3.3. Autophagy–Ferroptosis Crosstalk in Cellular Homeostasis

Both autophagy and ferroptosis are critical forms of programmed cell death that independently influence cellular physiology. These processes also interact significantly to regulate lipid, iron, and ROS metabolism [29]. Lipid droplets (LDs), which serve as the principal storage sites for triacylglycerols (TAGs), play a crucial role in managing energy stress within cells. Lipophagy is a selective form of autophagy characterized by the degradation of LDs. During this process, LDs are enveloped by autophagosome. Then, the TAGs within the LDs are hydrolyzed into fatty acids and glycerol, which are then released into the cytoplasm to serve as energy sources for the cell. Perilipin 2 (PLIN2) and perilipin 3 (PLIN3) located on the surface of LDs, contribute to the stability of LDs [30]. Upon knockout of PLIN2, lipophagy is enhanced [31]. Moreover, the degradation of PLIN2 and PLIN3 mediated by CMA can facilitate the hydrolysis of TGAs, thereby providing energy under glucose stress conditions, and the deficiency of CMA can result in lipid accumulation. Conversely, the biosynthesis of LDs is regulated by autophagy, through the degradation of membrane-containing organelles [32]. However, excessive autophagy may also contribute to the induction of ferroptosis [7]. Ferritinophagy mediated by nuclear receptor coactivator 4 (NCOA4), results in the degradation of ferritin and subsequently increases the levels of free iron within the cell. The inhibition or depletion of NCOA4 or autophagy-related proteins such as Atg3, Atg5, Atg7, and Atg13 leads to reduced ferritin degradation and free iron levels, thereby inhibiting ferroptosis [33]. On the one hand, free iron can generate ROS through the Fenton reaction and Haber–Weiss reaction, which can further promote lipid peroxidation and induce ferroptosis [21]. On the other hand, Fe (II) is part of the active composition of lipid oxidation enzymes such as LOX, also promoting lipid peroxidation and contributing to the induction of ferroptosis. As a crucial autophagy regulator, BECN1 can interact with the cystine transporter solute carrier family 7 member 11 (SLC7A11). SLC7A11 is responsible for exchanging extracellular cystine with intracellular glutamate at a 1:1 ratio, thereby influencing the biosynthesis of glutathione (GSH). The BECN1-SLC7A11 complex indirectly inhibits the function of SLC7A11, promoting ferroptosis. During this process, the sites of S90 and S93 of BECN1 are phosphorylated by AMPK, which facilitates the formation of the BECN1-SLC7A11 complex [34], while another crucial autophagy regulator, AMPK, is activated in response to energy stress. The activation of AMPK inhibits the biosynthesis of PUFAs, thereby alleviating sensitivity to ferroptosis [27]. In addition, the knockout of AMPK in a hepatocyte cell leads to an increase in hepcidin expression. This results in decreased levels of FPN, which in turn enhances the cell’s sensitivity to ferroptosis. Conversely, inhibition of AMPK can prevent the formation of this complex, thereby inhibiting ferroptosis [35]. Additionally, CMA can facilitate the degradation of GPX4 via HSP90, thereby promoting ferroptosis. Meanwhile, autophagy plays a critical role in clearing damaged organelles, such as mitochondria and endoplasmic reticulum, thus inhibiting the excessive generation of malondialdehyde and ROS [32].

**Figure 3:**
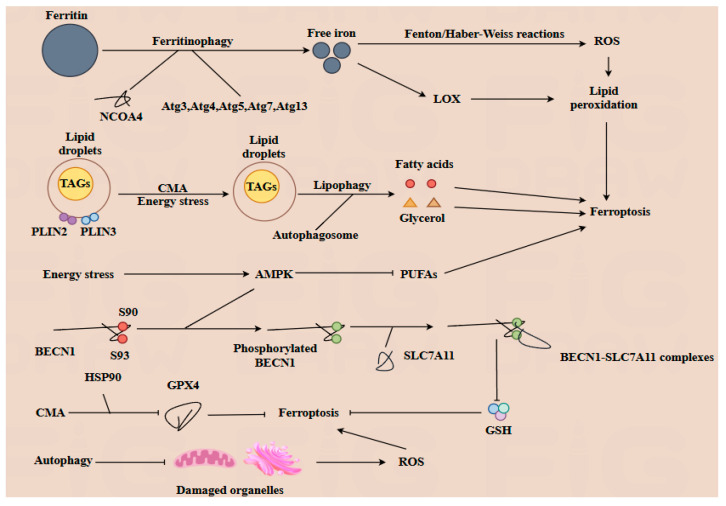
The interplay between autophagy and ferroptosis, two evolutionarily conserved cell death modalities, constitutes a sophisticated regulatory network involving multiple intersecting molecular nodes. Ferritinophagy, orchestrated by nuclear receptor coactivator 4 (NCOA4) in conjunction with core autophagy-related proteins (Atg3, Atg4, Atg5, Atg7, and Atg13), mediates ferritin degradation to release free iron into the cytosol. This liberated iron exerts dual pro-ferroptotic effects: (1) promoting reactive oxygen species (ROS) generation via Fenton/Haber–Weiss reactions, and (2) acting as an essential cofactor for lipoxygenases (LOXs) to amplify lipid peroxidation—a hallmark of ferroptotic execution. Under energy stress, chaperone-mediated autophagy (CMA) modulates lipid metabolism through phosphorylation of perilipin 2 (PLIN2) and perilipin 3 (PLIN3), triggering lipophagy to hydrolyze lipid droplets into free fatty acids and glycerol. This catabolic process attenuates lipid peroxidation by reducing substrate availability. Concurrently, AMP-activated protein kinase (AMPK), a central autophagy regulator, suppresses ferroptosis by downregulating polyunsaturated fatty acid (PUFA) biosynthesis, thereby limiting peroxidation-prone lipid pools. Post-translational modifications further fine-tune this crosstalk. Phosphorylation of BECN1 at Ser90/Ser93 enhances its binding capacity to the cystine/glutamate antiporter SLC7A11, forming a stabilizing complex that preserves glutathione (GSH) biosynthesis and redox homeostasis to antagonize ferroptosis. Conversely, CMA-mediated degradation of glutathione peroxidase 4 (GPX4), a master regulator of ferroptosis, compromises cellular antioxidant defenses, thereby sensitizing cells to ferroptotic death. Notably, macroautophagy exhibits paradoxical roles: while its capacity to eliminate ROS-generating damaged mitochondria indirectly suppresses ferroptosis, excessive organelle clearance may deplete endogenous antioxidants, creating context-dependent outcomes.

In conclusion, the interplay between autophagy and ferroptosis is intricate and multifaceted. Both autophagy and ferroptosis play pivotal roles in regulating numerous physiological processes, including embryonic and fetal development. Moreover, the two mechanisms can be modulated by gut microbiota and its metabolites, which will be further explored in subsequent sections (Figure 3).

## 4. Dynamic Interplay of Placental Autophagy and Ferroptosis in Pregnancy Physiology and Pathology

### 4.1. Autophagy in Placental Development

Autophagy plays a pivotal role in regulating embryonic and fetal development. The inhibition of autophagy at any stage may induce FGR [36]. During the blastocyst stage, autophagy, mediated by Atg7, promotes the transition of embryonic stem cells (ESCs) from the naive state to the primed state [37]. Notably, as the key transcription factor, Nanog homeobox protein maintains the differentiated function and inhibits the unnecessary differentiation of ESCs. The degradation of Nanog homeobox protein is regulated through SQSTM1/p62-mediated autophagy [38]. Furthermore, the decrease in autophagy leads to a higher level of apoptosis, a reduction in the number of blastocyst cells, and a significant decrease in the ratio of inner cell mass (ICM) to trophectoderm (TE) [39,40]. Moreover, the activation of autophagy in TE is higher than in ICM, suggesting that autophagy plays a crucial role in regulating the function of both TE and ICM [41]. Autophagy is highly activated during early pregnancy decidualization, promoting the adhesion and retention of NK cells in the decidua [42]. When autophagy is inhibited, the retention of individual NK cells decreases, leading to spontaneous abortion [6]. Uterine receptivity is critical for establishing and maintaining pregnancy [43]. For the endometrium to become receptive, stromal cells must differentiate into decidual cells capable of secreting factors necessary for embryo survival and placental development. In the absence of Atg16L1, endometrial stromal cells fail to decidualize properly, resulting in a reduced number of implantable blastocysts [44,45]. Before implantation, the embryo resides in a hypoxic environment with the activation of HIF-1α, which can further stimulate autophagy through epigenetic modification [39]. In the early stages of embryonic development, autophagy is involved in the apoptosis and defense mechanisms of the unfolded protein response to endoplasmic reticulum stress [46,47,48]. Additionally, autophagy contributes to upgrading mitochondrial fission and activity, thereby maintaining the dynamic balance of mitochondria [49]. Consequently, autophagy plays a crucial role in improving the developmental potential of the embryo. During the blastocyst development stage, the trophoblast stem cells differentiate into villous trophoblasts and extravillous trophoblasts (EVTs). Villous trophoblasts can be further divided into cytotrophoblasts and syncytiotrophoblasts (STBs) [6]. Nutrient deficiency and inhibition of mTORC1 can facilitate the fusion of cytotrophoblasts to form STBs. Additionally, autophagy provides energy for EVT invasion, playing a crucial role in EVT invasion and blood vessel remodeling. Intriguingly, the placenta with Atg7 gene knockout is smaller than the wild-type placenta, which further highlights the essential role of autophagy in placental development. As noted, the characteristics of the Atg7 gene knockout placenta include reduced trophoblast invasion and impaired vascular remodeling [46]. Autophagy also plays an essential role in regulating the differentiation of fetal organs. For instance, autophagy is highly activated in fetal neutral epithelial cells, and the inhibition of autophagy induces neural tube defects [46,50]. In the fetal digestive cells, autophagy-related genes are significantly activated between 6 and 9 weeks of gestation. In addition, defective autophagy during pregnancy leads to NLRP3 inflammasome activation, which promotes the secretion of IL-1β and IL-18. Therefore, autophagy regulates inflammation by clearing inflammasomes, thereby contributing to resistance against infection [51].

In conclusion, autophagy serves as a pivotal regulator in embryonic and fetal development, maintaining cellular homeostasis and ensuring proper placental function. Emerging evidence suggests that dysregulation of autophagy contributes significantly to pregnancy complications, such as GDM. The regulation of autophagy by gut microbiota and its metabolites, as well as the interplay between autophagy and ferroptosis during pregnancy, are discussed in the following sections.

### 4.2. Ferroptosis in Placental Development and Autophagy

Ferroptosis plays a dual role during pregnancy. On one hand, it is essential for proper placental formation by regulating trophoblast differentiation and placental development, which supports fetal growth and vascular remodeling. On the other hand, excessive or dysregulated ferroptosis has been implicated in various gestational complications, including PE, GDM, preterm birth, and FGR. Moreover, elevated levels of ROS, iron, and lipid peroxidation, alongside decreased levels of GPX4 in maternal serum and placental tissue compared to normal pregnancies, suggest that ferroptosis may contribute to adverse pregnancy outcomes [52,53]. During trophoblast invasion, the level of LC3-II and GPX4 is elevated, which indicates a higher frequency of autophagy. Autophagy plays a crucial role in clearing damaged organelles, such as mitochondria, thereby supplying energy to trophoblasts and inhibiting ferroptosis [54]. Additionally, the expression level of ACSL4 is deregulated. These changes suggest that ferroptosis is inhibited in trophoblasts [55]. During vascular remodeling, EVTs invade the maternal spiral arteries, progressively replacing the endothelial cells and degrading the smooth muscle cells. This transformation shifts the vasculature from a state of high blood resistance and low circulation to one of low blood resistance and high circulation [56,57]. During reperfusion, partial pressure of oxygen increases from approximately 15–20 mm Hg to 50 mm Hg [58]. The increased partial pressure of oxygen promotes the production of ROS in endothelial cells. Concurrently, the level of ACSL4 is upregulated in these cells. This suggests that ferroptosis plays a crucial role in clearing endothelial cells to facilitate EVT invasion and placental formation [59]. Moreover, ferroptosis in endothelial cells can propagate over long distances (≥5 mm) at constant speeds (approximately 5.5 μm/min) through trigger waves of ROS [60]. During the second and third trimesters of pregnancy, the demand for iron increases to 4–6 mg/day and during the final 6–8 weeks of pregnancy, this requirement further rises to 10 mg/day [61,62]. Additionally, during reperfusion, HIF-1α enhances mitophagy through the BNIP3 pathway to clear damaged mitochondria, thereby inhibiting excessive ferroptosis. Maternal hepcidin secretion decreases during the second and third trimesters, potentially due to the influence of unknown hormones produced by the placenta or fetus [63]. Hepcidin typically binds to FPN on the cell membrane surface, inhibiting iron export from cells, which would otherwise lead to a lower concentration of serum iron. However, during pregnancy, the reduced hepcidin levels facilitate increased iron availability for fetal needs [61,64]. Additionally, if the storage of iron is deficient, the NCOA4-mediated ferritinophagy may be activated to support the fetal demand for iron. However, the specific regulatory mechanisms still require further investigation [65]. STB, as the primary interface between mother and fetus, plays a crucial role in maintaining placental function. TFR1 is widely expressed in various cell types, with particularly high levels in STB. The elevated expression of hepcidin and TFR1 ensures that STB can unidirectionally absorb iron to meet the fetal iron requirements [8]. Notably, even under conditions of physiological iron deficiency, iron is still enriched in the STB. This characteristic increases the vulnerability of STB to oxidation stress. Actually, STB is more susceptible to ROS damage and ferroptosis [52]. Concurrently, excessive ROS and unfolded protein response can activate mitophagy and reticulophagy, thus alleviating ferroptosis within STB, thereby preventing adverse pregnancy outcomes such as PE [66]. The rate of lipolysis increases during the second and third trimesters of pregnancy to meet the heightened energy demands of fetal development. However, this increase can elevate the risk of ferroptosis. This period is also characterized by a higher incidence of pregnancy complications. Excessive ferroptosis is often associated with these gestational syndromes, indicating that its over-occurrence is significantly linked to adverse pregnancy outcomes [67]. The levels of Nrf2, SLC7A11, and GPX4 are reduced in placental tissue from patients with PE, indicating a diminished resistance to oxidative stress. Conversely, the levels of ferroptosis-related substances such as MDA, ACSL4, ALOX-5, NOX-1, and NOX-2 are elevated compared to the normal group [68]. Additionally, studies showed that Atg-knockout placentas are significantly smaller than those in wild-type mice. Furthermore, reduced TFEB levels in PE impair autophagy flux, leading to placental oxidative stress and ferroptosis, which further exacerbate PE pathogenesis. These molecular alterations synergistically increase placental vulnerability to ferroptosis-driven and autophagy-deficient pathways, ultimately accelerating PE progression [46,69,70,71]. In patients with GDM, excessive insulin resistance promotes the production of endogenous glucose, which can further exacerbate fatty acid degradation, leading to disorders in both glucose and lipid metabolism [72]. Meanwhile, the expression level of GPX4 is lower than in the normal group, while the expression of ACSL4 is increased in placental tissue, which leads to PUFA-PL synthesis, thereby promoting the occurrence of ferroptosis in placental tissue [71]. Additionally, the levels of ATG5, Beclin-1, LC3-II, and p62 are lower compared with healthy pregnancies, suggesting that the excessive ferroptosis and inhibited autophagy can potentially contribute to GDM [73]. A comparison between preterm birth and artificial abortion in trophoblasts reveals that the level of GPX4 is reduced in patients experiencing preterm birth, suggesting a potential association between ferroptosis and preterm birth [2]. Meanwhile, decreased levels of ATG16L1 and LC3 compared with healthy pregnancies suggest that excessive ferroptosis activation and impaired autophagy may be contributing factors to preterm birth [74]. However, further evidence is required to substantiate this link.

In conclusion, whereas ferroptosis exhibits a positive role in vascular remodeling, it exacerbates pregnancy complications during the second and third trimesters. The intricate interplay between autophagy and ferroptosis warrants further investigation to clarify how their interaction impacts maternal and fetal health, particularly in the context of pregnancy-related disorders. Notably, ferroptosis markers, particularly ACSL4, emerge as promising diagnostic targets for monitoring and potentially predicting pregnancy complications. However, additional clinical data are required to validate their efficacy and establish robust diagnostic protocols.

## 5. Gut Microbiota–Metabolite Axis Orchestrates Placental Autophagy–Ferroptosis Balance in Pregnancy

Gut microbiota and their metabolites play a dual regulatory role in modulating placental autophagy and ferroptosis during pregnancy. Key metabolites such as SCFAs, lipopolysaccharide (LPS), TMAO, tryptophan (Trp) derivatives, and bile acid derivatives are intricately linked pregnancy outcomes [9,75,76,77,78]. SCFAs, predominantly acetic acids, propionic acids and butyric acids, constitute 90–95% of intestinal metabolites. During pregnancy, acetic acid elevates the AMP/ATP ratio, upregulates LKB1 expression, and promotes autophagy, ensuring the placental tissue stability. Propionic and butyric acids enhance histone H3 acetylation at the ULK1 promoter, activating the AMPK signaling pathway in the placenta, thereby protecting placental and fetal development. Butyric acid activates the SLC7A11/TXNRD1/GPX4/Nrf2 signaling pathway to inhibit ferroptosis [75,79]. Furthermore, SCFAs inhibit the secretion of anti-inflammatory cytokines such as IL-6, TNF-α, and IL-1β, thereby reducing ROS production. Thus, SCFAs play a significant role in protecting ovarian, placental, and fetal development [75,80]. Since excessive ferroptosis in trophoblasts and placental tissue may induce PE, the regulation of ferroptosis mediated by SCFAs is crucial for maintaining healthy pregnancy outcomes [9,75,81]. LPS, produced by Gram-negative bacteria, activates the expression of the TLR4/NF-κB and MAPK pathways, leading to the secretion of pro-inflammatory cytokines such as IL-6, TNF-α, and IL-1β. This inflammatory response promotes the depletion of GSH and downregulates the expression of GPX4, thereby inducing ferroptosis in placental tissue [77]. Concurrently, excessive LPS destroys the intestinal barrier, resulting in gut leakage, which further increases the systemic circulation of LPS and enhances placental inflammation. Studies have demonstrated that LPS-induced inflammation can trigger placental inflammation and ferroptosis. Notably, SCFAs were shown to alleviate the damage caused by LPS, providing a potential therapeutic avenue for alleviating LPS-induced injury [76,77,82,83]. Trimethylamine, a metabolite derived from gut microbiota, can be oxidized to TMAO. TMAO activates the MAPKs/Nrf2-keap1 pathway, leading to the generation of ROS and iron accumulation. Concurrently, ROS inhibits the expression of GPX4 as well. Furthermore, TMAO activates the NF-κB pathway, which upregulates the production of pro-inflammatory cytokines such as IL-6 and TNF-α, thereby exacerbating placental and systemic inflammatory response. Additionally, TMAO-induced calcium mobilization triggers the unfold protein response, further promoting inflammation. Elevated levels of TMAO can induce vascular endothelial injury and trophoblast dysfunction, as well as accelerate insulin resistance and glucose metabolism disorders, particularly in patients with PE and GDM [77,84,85,86]. Gut microbiota can convert Trp into indole derivatives. Both Trp and its derivatives play a significant role in regulating autophagy and ferroptosis. For example, after Trp or its derivatives bind to an aryl hydrocarbon receptor, the secretion of IL-6 is inhibited. Moreover, these derivatives can upregulate ALDH1A3, an enzyme that promotes the production of NADH, thereby reducing oxidative stress. ALDH1A3 also positively influences the production of coenzyme Q10, mediated by FSP1, which is essential for cellular antioxidant defense. Kynurenine is involved in the biosynthesis of 3-hydroxykynurenine and 5-hydroxytryptamine, which are derived from Trp; they play a positive role in clearing free radical and inhibiting lipid oxidation. Furthermore, Trp and its derivatives can enhance the AMPK/SIRT1 pathway, thus further enhancing autophagy and alleviating ferroptosis to protect placental and fetal development. Additionally, Trp and its derivatives inhibit NF-κB activation induced by LPS, thereby alleviating LPS-induced placental inflammation [13,87,88]. Cholic Acid is an important endogenous bile acid, exhibiting a weaker binding capability with farnesoid X receptor (FXR). However, the gut microbiota can convert cholic acid into DCA, which has a better ability to bind with FXR. After bile acids bind to FXR, the expression level of anti-ferroptotic genes, including GPX4, FSP1, and PPARα are upregulated, thereby effectively inhibiting ferroptosis [13,89].

In conclusion, gut microbiota and its metabolites can regulate the inflammatory response to indirectly modulate ferroptosis and autophagy and directly regulate autophagy- and ferroptosis-related signaling pathways during pregnancy. Importantly, gut microbiota is a critical determinant of pregnancy outcomes, as its dysregulation is frequently implicated in pregnancy complications that adversely affect placental and fetal development. Notably, the composition of gut microbiota and its metabolites may serve as novel diagnostic biomarkers for identifying pregnancy complications during pregnancy. However, this therapeutic strategy requires further exploration (Table 1).

## 6. Translational Applications: From Mechanisms to Precision Medicine

**Figure 4:**
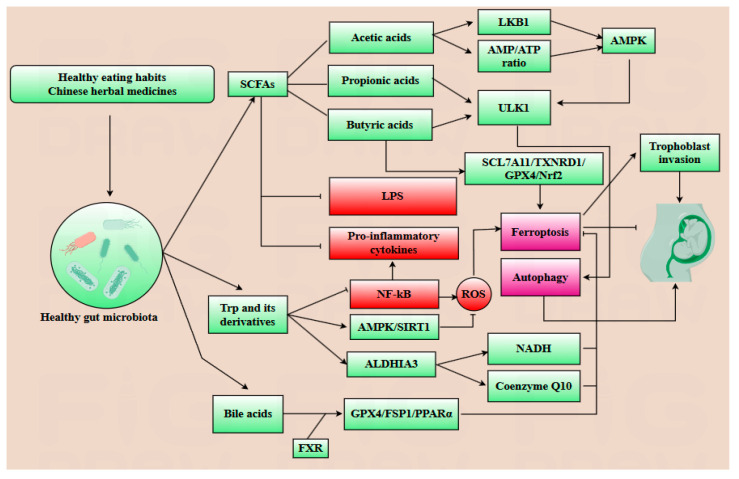
Healthy dietary patterns and Chinese herbal medicines foster a favorable gut microbiota, which orchestrates the biosynthesis of short-chain fatty acids (SCFAs), tryptophan (Trp) derivatives, and bile acids. Acetic acid upregulates LKB1 and elevates the AMP/ATP ratio, thereby activating AMPK. Meanwhile, propionic and butyric acids promote the ULK1 complex assembly. Collectively, activated AMPK and ULK1 synergistically boost autophagic activity. Additionally, butyric acid suppresses ferroptosis by upregulating the SLC7A11/TXNRD1/GPX4/Nrf2 axis. SCFAs also exert dual anti-ferroptotic effects: they dampen lipopolysaccharide (LPS)-induced pro-inflammatory cytokine production and mitigate ROS accumulation, thereby attenuating ferroptosis. Trp derivatives suppress NF-κB activation, consequently reducing pro-inflammatory cytokine secretion and blunting ferroptosis. Concurrently, they augment AMPK/SIRT1 signaling, which reduces ROS production and further suppresses ferroptosis. Moreover, ALDHA3 upregulation fuels NADH and coenzyme Q10 biosynthesis, fortifying antioxidant defenses against ferroptosis. Bile acids, particularly deoxycholic acid (DCA), exhibit higher farnesoid X receptor (FXR) binding affinity than primary counterparts (e.g., cholic acid, CA), activating the GPX4/FSP1/PPARα axis to suppress ferroptosis.

**Figure 5:**
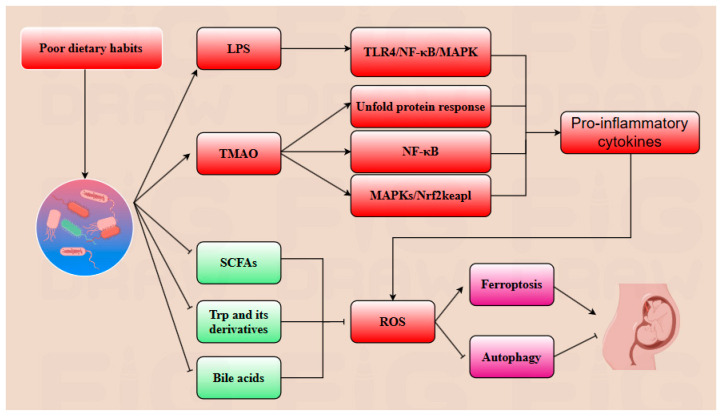
Poor dietary habits foster the overgrowth of a pathogenic gut microbiota, which synthesizes deleterious metabolites including lipopolysaccharide (LPS) and trimethylamine N-oxide (TMAO). LPS elicits TLR4/NF-κB/MAPK signaling cascades, fueling pro-inflammatory cytokine production. TMAO induces an unfolded protein response, activating endoplasmic reticulum stress, and it perturbs Nrf2-Keap1 redox homeostasis—thereby elevating reactive oxygen species (ROS) and lipid peroxidation. Concomitantly, the dysbiotic microbiota suppresses the biosynthesis of beneficial metabolites (short-chain fatty acids (SCFAs), tryptophan (Trp) derivatives, and bile acids), exacerbating ROS accumulation. This dual hit synergistically triggers ferroptosis while suppressing cytoprotective autophagy, ultimately culminating in adverse pregnancy outcomes.

The specific composition of gut microbiota and its metabolites have emerged as promising therapeutic targets for alleviating abnormal placental autophagy and ferroptosis during pregnancy, as well as potential diagnostic indicators for pregnancy-related complications [98]. Plenty of traditional Chinese medicines have demonstrated their effects on modulating gut microbiota composition. For example, SCM-198, the active component of Leonurus, has been shown to enhance the population of butyrate-producing bacteria, particularly *C. minuta*, while directly regulating the SLC7A11/GPX4 axis and activating the Nrf2 pathway to inhibit excessive ferroptosis during pregnancy [84]. Diammonium glycyrrhizinate, the principal constituent of licorice, exhibits multiple regulatory benefits on gut microbiota by reducing the abundance of endotoxin-producing bacteria such as *Desulfovibrio* and promoting the growth of SCFA-producing bacteria, particularly *Lactobacillus* species to alleviate the abnormal autophagy and ferroptosis during pregnancy [99]. Dan Shen demonstrated positive regulatory effects on gut microbiota composition as well, specifically increasing the abundance of *Akkermansia*, *A. muciniphila*, and *Lactobacillus* in spontaneously hypertensive rats models, implicating the potential effect on mitigating abnormal placental autophagy and ferroptosis during gestation [12]. Dietary habits play a pivotal role in modulating the composition of gut microbiota, with certain beneficial dietary patterns exerting positive regulatory effects. The consumption of probiotic-rich foods, particularly those containing *Lactobacillus* fermentum and *Bifidobacterium*, has been demonstrated to alleviate insulin resistance and lipid metabolic disorders. Prebiotics such as inulin also contribute significantly to the favorable regulation of gut microbiota composition. Following oral administration of inulin, fecal analysis via PCR revealed a marked upregulation of *Faecalibacterium prausnitzii* and *Bifidobacterium* levels [100,101]. Additionally, the intake of fish oil has been associated with an increase in *Bifidobacterium* abundance, and a reduction in *Haemophilus parainfluenzae* and *Veillonella parvula* populations. Furthermore, the intake of fiber-rich foods has been shown to enhance the abundance of *Roseburia* and *Lachnospiraceae*, thereby promoting the production of SCFAs and indole, which are crucial for maintaining the process of pregnancy [102,103]. A high-protein diet has been shown to decrease the abundance of *Proteobacteria* while promoting the growth of methane-producing bacteria. These methane-producing bacteria play a crucial role in fermenting dietary fiber to produce SCFAs and converting trimethylamine into methane, thereby mitigating the damage induced by TAMO. Conversely, poor dietary habits have been implicated in dysregulating gut microbiota, leading to abnormal autophagy and ferroptosis during pregnancy. For instance, a high-sugar diet is associated with reduced production of indoles whereas low-fiber and high-fat diets correlate with decreased gut microbial α-diversity [104]. Notably, excessive fruit consumption may diminish the enrichment of beneficial bacteria such as *Lactobacillus* and *Bacteroides*, due to its high fructose content. The gut microbiota and pregnancy share a dynamic, mutual regulatory relationship, where changes in the composition of gut microbiota and its metabolites can serve as diagnostic markers for pregnancy progression. For instance, in patients with PE, the abundance of *Bifidobacterium* is decreased, which is associated with reduced biosynthesis of SCFAs and impaired gut barrier function. These changes contribute to heightened inflammatory responses in the placenta [12,82]. Conversely, the levels of *Klebsiella*, *Gemmobacter*, and *Ruegeria* are increased in PE, leading to elevated expression of precursors of TMAO such as betaine, creatinine, and L-carnitine. This elevation influences the levels of ROS, inhibits autophagy, and induces ferroptosis, thereby exacerbating placental dysfunctions and pregnancy complications [105]. In conclusion, gut microbiota α-diversity is significantly reduced in complicated pregnancies, resulting in alterations in metabolites that contribute to abnormal placental autophagy and ferroptosis [106,107]. Thus, both the fecal microbiota composition and blood metabolites can serve as potential diagnostic indicators [106]. However, the diagnostic utility of gut microbiota requires further exploration due to its high variability influenced by dietary habits, age, and lifestyle. Consequently, the development of a reliable gut microbiota-based diagnostic approach demands extensive research and substantial data [82]. Notably, prior to delivery, the abundance of inflammation-inducing bacteria increases, potentially amplifying the inflammatory response and facilitating labor onset. Additionally, ferroptosis levels may rise before delivery, while autophagy activity decreases. Nevertheless, further investigation is needed to clarify whether ferroptosis plays a significant regulatory role in the delivery process (Figure 4 and Figure 5).

## 7. Conclusions and Future Perspectives

Gut microbiota-derived metabolites exert dual regulatory effects on placental autophagy and ferroptosis during pregnancy, with their actions exhibiting trimester-specific spatiotemporal dynamics. Key metabolites such as SCFAs and TMAO modulate critical nodes in these pathways including AMPK, ACSL4, and GPX4. The dynamic flux between autophagy and ferroptosis critically influences placental development, thereby determining pregnancy outcomes such as PE and FGR. Current studies predominantly focus on pairwise interactions, such as gut microbiota metabolites and autophagy, gut microbiota metabolites and pregnancy complications, or interplay between placental autophagy and ferroptosis. However, the integrated axis encompassing gut microbiota, their metabolites, placental autophagy, and ferroptosis during pregnancy remains underexplored. It is noteworthy that, although necroptosis, pyroptosis, and senescence are linked to placental ROS stress, autophagy and ferroptosis are distinguished by two key features: (1) they are uniquely modulated by microbial metabolites at the nutrient-sensing nodes (e.g., AMPK/mTOR for autophagy, GPX4 for ferroptosis), forming a “microbiota–metabolite-cell death” regulatory axis; (2) they exhibit dynamic, trimester-specific patterns in placenta, a temporal specificity less characterized in other cell death pathways. Thus, this mechanistic uniqueness and gestational relevance support their prioritization in this review. Key unresolved issues among the integrated axis include the following: (1) The threshold dynamics and gestational specificity (e.g., the metabolites thresholds required to trigger autophagy or ferroptosis trophoblasts invasion). (2) Hormonal feedback mechanisms (e.g., investigating whether ferroptosis inhibition in placental tissue upregulated pregnancy-maintaining hormones, or determining if progesterone production indirectly suppresses ferroptosis, since progesterone plays a positive role in maintaining pregnancy). (3) Tissue- and organ-specific metabolite effects—since metabolites influence all tissues and organs, the interplay between these systems and the placenta/fetus requires further exploration. Notably, elucidating this integrated axis contributes to developing diagnostic and therapeutic strategies for pregnancy complications. Potential diagnostic innovations include multi-omics panels integrating maternal serum GPX4 activity, placental ACSL4/LC3B immunohistochemistry, and fecal SCFA-TMAO ratios for early prediction of gestational disorders. Additionally, therapeutic advances may involve engineered probiotics and metabolite-targeted interventions to mitigate pregnancy-related pathologies.

## Figures and Tables

**Table 1:** Summarizing the metabolites’ effects on ferroptosis and their key reaction mechanisms.

Metabolites	Influence	Key Reaction	Reference
PLOOH	Promotion	Reacts with iron(II) or iron(III) to generate lipid alkyl radicals and lipid peroxyl radicals, which further react with PUFA to generate more PLOOH.	[90,91]
ROS	Promotion	Promotes lipid oxidation, thereby further enhancing the degradation of FPN.	[23]
ACSL4	Promotion	Catalyzes the conversion of PUFA to acyl-CoA, which is further incorporated into phospholipids.	[92]
Free iron	Promotion	Facilitates the conversion of PUFA to PLOOH in the cell membrane and promotes ROS production via the Fenton and Haber–Weiss reactions.	[93]
LPS	Promotion	LPS promotion promotes secretion of pro-inflammatory cytokines and ROS by activating TLR4/NF-κB/MAPK pathways.	[77]
TMAO	Promotion	Promotes the unfolded protein response and upregulates NF-κB/MAPKs/Nrf2-Keap1 pathways.	[86]
GPX4	Inhibition	Utilizes reduced GSH to detoxify harmful lipid peroxides into non-toxic lipid alcohols and scavenges ROS.	[94]
Vitamin E	Inhibition	Neutralizes free radicals on the plasma membrane, thereby preventing peroxidation of PUFAs in the membrane.	[95]
CoQ10H2	Inhibition	Neutralizes free radicals and scavenges accumulated lipid peroxides, thereby alleviating oxidative damage.	[95]
NADPH	Inhibition	Supplies a large amount of reducing equivalents in the form of hydrogen in cells, and plays a key role in reducing oxidized antioxidants back to their reduced state.	[96]
FSP1	Inhibition	Upregulates the NADP/NADPH ratio and utilizes reducing equivalents to reduce CoQ10 back to CoQ10H2.	[97]
SCFAs	Inhibition	Promote ULK1 synthesis, thereby further upregulating the autophagy pathway to inhibit ferroptosis.	[81]
Trp and its derivatives	Inhibition	Upregulate AMPK/SIRT1 and ALDH1A3 pathways to inhibit ROS production, thereby suppressing ferroptosis.	[87]
Bile acids	Inhibition	Upregulate the GPX4/FSP1/PPARα axis, inhibit ROS generation, thereby further suppressing ferroptosis.	[20]

## Abbreviations

ACSL4	Long-chain acyl-coenzyme A synthase 4
AMPK	AMP-activated protein kinase
CDCA	Chenodeoxycholic acid
CMA	Chaperone-mediated autophagy
DCA	Deoxycholic acid
ESCs	Embryonic stem cells
EVTs	Extravillous trophoblasts
FGR	Fetal growth restriction
FPN	Ferroportin
FSP1	Ferroptosis suppression protein 1
FXR	Farnesoid X receptor
GDM	Gestational diabetes mellitus
GPX4	Glutathione peroxidase 4
GSH	Glutathione
HSC70	Heat-shock cognate protein 70
HSP	Heat shock protein
ICM	Inner cell mass
LAMP-2A	Lysosome-associated membrane protein type 2A
LC3	Microtubule-associated protein 1 light chain 3
LDs	Lipid droplets
LPS	Lipopolysaccharide
NCOA4	Nuclear receptor coactivator 4
PE	Preeclampsia
PI	Phosphatidylinositol
PI3P	Phosphatidylinositol 3-triphosphate
PLIN2	Perilipin 2
PLIN3	Perilipin 3
PLOOH	Phospholipid hydroperoxides
PUFA	Polyunsaturated fatty acids
ROS	Reactive oxygen species
SCFAs	Short-chain fatty acids
SLC7A11	Cystine transporter solute carrier family 7 member 11
STBs	Syncytiotrophoblasts
TAGs	Triacylglycerols
TE	Trophectoderm
TMAO	Trimethylamine N-oxide
Trp	Tryptophan
